# The effect of antacid and mineral supplements on bictegravir pharmacokinetics: results from a Phase 1, open-label, drug–drug interaction study

**DOI:** 10.1128/aac.00781-25

**Published:** 2025-11-28

**Authors:** Priyanka Arora, Jason T. Hindman, Steve West, John Ling, Ramesh Palaparthy, Dhananjay D. Marathe

**Affiliations:** 1 Gilead Sciences, Inc. 2158 , Foster City, California, USA; Houston Methodist Hospital and Weill Cornell Medical College, Houston, Texas, USA

**Keywords:** bictegravir, pharmacokinetics, drug–drug interactions, metal cations, human immunodeficiency virus, integrase strand transfer inhibitor

## Abstract

The mechanism of action of integrase strand transfer inhibitors involves binding to magnesium ions in the active site of the HIV integrase enzyme, making them susceptible to chelation-type drug–drug interactions with metal cation–containing medications. This study evaluated the potential of metal cation–containing antacids and mineral supplements to impact bictegravir (BIC) exposure and assessed alternative approaches for combined use. This was an open-label, single-dose, Phase 1 study in adult participants without HIV. The pharmacokinetics and safety of BIC (administered as part of a single-tablet combination with emtricitabine [F] and tenofovir alafenamide [TAF; B/F/TAF]) were assessed when co-administered with maximum-strength aluminum/magnesium-containing antacid (referred to as “aluminum/magnesium-containing antacid”), calcium carbonate, or ferrous fumarate under fasted and fed conditions, and administered 2 hours before or after the antacid. Pharmacokinetic parameters were compared using analysis of variance to calculate geometric least-squares mean ratios and 90% confidence intervals. Forty-two participants were enrolled. BIC exposure (area under the plasma concentration–time curve extrapolated to infinity) was reduced by 79%, 33%, and 63%, respectively, when co-administered with aluminum/magnesium-containing antacid, calcium carbonate, and ferrous fumarate under fasted conditions. Co-administration of B/F/TAF with calcium carbonate or ferrous fumarate with a meal and administration of B/F/TAF 2 hours before the antacid reduced the impact of the interactions. B/F/TAF was well tolerated alone or in combination with metal cation–containing medications. Co-administration of BIC and calcium/iron-containing supplements with a meal and administration of BIC 2 hours or more before aluminum/magnesium-containing antacids are some of the effective strategies to mitigate chelation effects on BIC exposure.

This study was registered at NCT05502341/NCT06333808.

## INTRODUCTION

Integrase strand transfer inhibitors (INSTIs) are recommended as part of the first-line treatment for people with HIV (PWH) (1, 2). Bictegravir (BIC) is a potent INSTI approved for the treatment of HIV-1 infection as part of an oral, once-daily, fixed-dose, single-tablet regimen, B/F/TAF, which also incorporates two nucleoside reverse transcriptase inhibitors, emtricitabine (F) and tenofovir alafenamide (TAF) (1–3). Clinical trials (4–7) and real-world studies (8, 9) have shown B/F/TAF to be effective and well tolerated in PWH. In addition, BIC is currently in Phase 2/3 clinical development in combination with lenacapavir, an HIV-1 capsid inhibitor (NCT05502341/NCT06333808) (10).

A key element of INSTI action involves chelation of magnesium ions in the active site of the HIV integrase enzyme, displacing the HIV viral DNA and preventing insertion into the host cell genome (11). Therefore, INSTIs, including BIC, are susceptible to chelation-type drug–drug interactions (DDIs) with metal cation–containing therapeutic products (12). Previous studies have shown reductions in the exposure of other INSTIs when co-administered with metal cation–containing therapeutic products (13–17).

PWH often take metal cation–containing medications in addition to antiretroviral therapy (ART); for example, gastroesophageal reflux and other gastrointestinal disorders are common among PWH, leading to frequent use of gastric acid modifiers, including antacids (18). Furthermore, HIV-1 infection and ART are associated with reductions in bone mineral density (19), and calcium supplements may be taken by PWH to improve bone health. PWH may also use iron supplements as they are at high risk of iron-deficiency anemia due to infection, chronic inflammation, and the effects of ART (20). Finally, as for the general population, PWH may also take general multivitamin/multimineral supplements containing metal cations (21).

This study evaluated the potential of metal cation–containing antacids and mineral supplements to impact BIC exposure in adult participants without HIV and assessed alternative approaches for combined use if an interaction was observed.

## MATERIALS AND METHODS

### Study design and participants

Study GS-US-380-3909 was an open-label, single-dose, fixed-sequence, multiple-cohort, multiple-period, adaptive Phase 1 study conducted in adult participants without HIV (typically referred to as healthy volunteers) at a single center within the United States between April 4, 2016, and May 20, 2016. Eligible participants were adult (18–45 years) males and non-pregnant, non-lactating females in good general health at a screening evaluation performed no more than 28 days before the first study dose. Key inclusion criteria included body mass index ≥19.0 and ≤30.0 kg/m^2^; normal 12-lead electrocardiogram (ECG) or clinically insignificant ECG abnormalities (as judged by the study investigator); normal renal function; estimated glomerular filtration rate using the Cockcroft-Gault method (22) ≥90 mL/min, based on serum creatinine and actual body weight; and no significant medical history. Full eligibility criteria are available in the supplement. All prescription and over-the-counter medications except vitamins, acetaminophen, ibuprofen, and hormonal contraceptives were not permitted during study participation.

The primary objectives were to evaluate the effects of simultaneous co-administration of aluminum/magnesium-containing antacid, or calcium or iron supplements, with B/F/TAF fixed-dose combination (FDC) under fasted and fed conditions on the pharmacokinetics (PK) of BIC. Additionally, the study aimed to compare the PK of BIC between staggered administration of the antacid and B/F/TAF compared with B/F/TAF FDC alone, under fasted conditions. The secondary objective was to evaluate the safety and tolerability of single doses of B/F/TAF FDC administered alone or in combination with aluminum/magnesium-containing antacid, or calcium or iron supplements.

Three dosing cohorts were evaluated (Table 1). All cohorts were initiated in parallel and received a single dose of B/F/TAF (50/200/25 mg) FDC as one tablet administered orally on Day 1 under fasted conditions, followed by a 7-day washout period. This was followed by co-administration of single doses of B/F/TAF with single doses of aluminum/magnesium-containing antacid, or calcium or iron supplements on Days 9, 17, and 25, respectively (Cohorts 1 and 3 only), with all treatments separated by a 7-day washout period. Vitamins and/or other supplements were not permitted on the dosing day.

**TABLE 1:** Study drug treatments within each cohort^*a*^

Cohort	Schedule							
	Day 1	Days 2–8	Day 9	Days 10–16	Day 17	Days 18–24	Day 25	Day 29
**Cohort 1** (fasted; simultaneous co-administration)	Single-dose B/F/TAF	Washout	Single-dose B/F/TAF and aluminum/magnesium-containing antacid^*b*^	Washout	Single-dose B/F/TAF and calcium carbonate 1,200 mg	Washout	Single-dose B/F/TAF and ferrous fumarate 324 mg	Discharge
**Cohort 2** (fasted; staggered administration)	Single-dose B/F/TAF	Washout	Single-dose B/F/TAF 2 h *before* aluminum/magnesium-containing antacid^*b*^	Washout	Single-dose B/F/TAF 2 h *after* aluminum/magnesium-containing antacid^*b*^	Discharge^*c*^		
**Cohort 3** (fed; simultaneous co-administration with food)	Single-dose B/F/TAF (under fasted conditions)	Washout	Single-dose B/F/TAF and aluminum/magnesium-containing antacid^*b*^	Washout	Single-dose B/F/TAF and 1,200 mg calcium carbonate	Washout	Single-dose B/F/TAF and 324 mg ferrous fumarate	Discharge

^*a*^ B/F/TAF, bictegravir/emtricitabine/tenofovir alafenamide (50/200/25 mg); h, hours.

^*b*^ 1,600 mg aluminum hydroxide/1,600 mg magnesium hydroxide/160 mg simethicone.

^*c*^ Discharge on Day 21.

Cohorts 1 and 3 were designed to assess the effect of single-dose antacid, calcium, or iron supplements simultaneously co-administered with single-dose B/F/TAF under fasted (Cohort 1) or fed (Cohort 3) conditions. Participants received maximum-strength antacid oral suspension (four teaspoons; 20 mL; 1,600 mg aluminum hydroxide/1,600 mg magnesium hydroxide/160 mg simethicone; referred to as “aluminum/magnesium-containing antacid”) on Day 9, calcium carbonate (1,200 mg; 2 × 600 mg tablets) on Day 17, and ferrous fumarate (1 × 324 mg tablet) on Day 25. Cohort 2 assessed the effect of staggered administration of single-dose, aluminum/magnesium-containing antacid oral suspension with single-dose B/F/TAF under fasted conditions. Participants received B/F/TAF 2 hours before the antacid on Day 9 and 2 hours after the antacid on Day 17. If the 2-hour separation window was found to be insufficient to minimize the interaction with BIC, a fourth, adaptive cohort was planned to evaluate the effect of 4-hour staggered administration of B/F/TAF and aluminum/magnesium-containing antacid.

A fixed dose of 50/200/25 mg B/F/TAF was chosen as this was the FDC formulation selected for Phase 3 clinical development (23). The highest recommended dose of aluminum/magnesium-containing antacid (24) was chosen because of its high divalent metal cation content, to evaluate the worst-case scenario for interaction. Commonly recommended daily doses for a calcium supplement (calcium carbonate) and an iron supplement (ferrous fumarate) were chosen, with elemental calcium and iron content of approximately 480 mg and 107 mg, respectively (25, 26). Given the half-life for BIC of ~17 hours (3), a crossover study with a 7-day washout between periods was considered appropriate.

Participants were confined to the clinic for the duration of the study, from Day −1 until completing study procedures on Day 29 for Cohorts 1 and 3, or Day 21 for Cohort 2. Follow-up phone calls were conducted 7 (± 2) days after clinic discharge to collect information on any adverse events (AEs) that may have occurred and any concomitant medications taken since discharge.

On Day 1, all participants received B/F/TAF administered following an observed overnight fast (for at least 10 hours). Subsequently, participants in Cohort 1 received study drugs on Days 9, 17, and 25, following an observed overnight fast; participants in Cohort 2 received study drugs on Days 9 and 17, following an observed overnight fast; and participants in Cohort 3 received study drugs on Days 9, 17, and 25, following an observed overnight fast and within 5 minutes of the participant finishing a moderate-fat breakfast (~600 kcal; ~27% fat).

### BIC PK analyses

Serial blood samples for PK assessment were taken on Days 1, 9, and 17 for Cohorts 1–3 and additionally on Day 25 for Cohorts 1 and 3 pre-B/F/TAF administration, and at 0.5, 1, 1.5, 2, 3, 4, 6, 8, 12, 18, 24, 36, 48, 72, and 96 hours post-dose. Concentrations of BIC in plasma samples were determined by QPS, LLC (Newark, DE) using fully validated liquid chromatography–tandem mass spectroscopy bioanalytical methods. All samples were analyzed within the timeframe determined using frozen stability storage data. The lower and upper limits of quantification for BIC were 0.020 µg/mL and 20 µg/mL, respectively. The interassay precision (coefficient of variation) and accuracy (relative error) ranges for BIC were 3.9%–5.7% and 2.4%–5.8%, respectively.

PK parameters were estimated using Phoenix WinNonlin software (Version 6.4, Pharsight Corporation, Princeton, NJ, USA) using standard noncompartmental analysis methods. Assessed key PK parameters included area under the plasma concentration–time curve extrapolated to infinity (AUC_inf_), plasma concentration after 24 h (C_24_), and maximum observed plasma concentration (C_max_).

The BIC PK analysis set included all enrolled participants who received at least one dose of the study drug and had at least one post-dose PK concentration value.

### Safety analyses

Assessments included periodic clinical laboratory tests, ECGs, and physical examinations (including vital signs) at Day −1 and/or Day 1, then every 8 days and at the end of the study (Day 29 for Cohorts 1 and 3, and Day 21 for Cohort 2), and daily monitoring of AEs and concomitant medications.

The safety analysis set included all participants who received at least one dose of the study drug.

### Statistical analyses

Participant demographics and baseline characteristics were summarized using descriptive statistics.

Plasma concentrations and PK parameters for BIC were summarized using descriptive statistics, by treatment within each cohort.

AUC_inf_, C_24_, and C_max_ for test treatments (B/F/TAF plus study drug) were compared with reference treatment (B/F/TAF alone under fasted or fed conditions) using mixed-effects analysis of variance to calculate geometric least-squares mean (GLSM) ratios and associated 90% confidence intervals (CIs). The estimated 90% CIs were compared to a prespecified lack of DDI boundary of 70%–143%. Previously reported clinical data support the use of a 70%–143% boundary over the traditional 80%–125% bioequivalence boundary for evaluation of quantitative significance for DDI effects; BIC has a long dissociation half-life (~163 hours), a flat exposure–response curve for efficacy, and no additional safety concerns over a broad dose/exposure range (27–31). Generally, test drugs with 90% CIs entirely contained within this range were deemed to have no significant effect on the PK of BIC. A minimum of 12 evaluable participants in each cohort was calculated to be needed to reject the null hypothesis (i.e., the 90% CI for the GLSM ratio for each PK parameter was outside the prespecified boundary) with ≥90% power.

AEs, laboratory parameters, graded laboratory abnormalities, and vital signs were summarized using descriptive statistics.

## RESULTS

### Study participants

**Fig 1:**
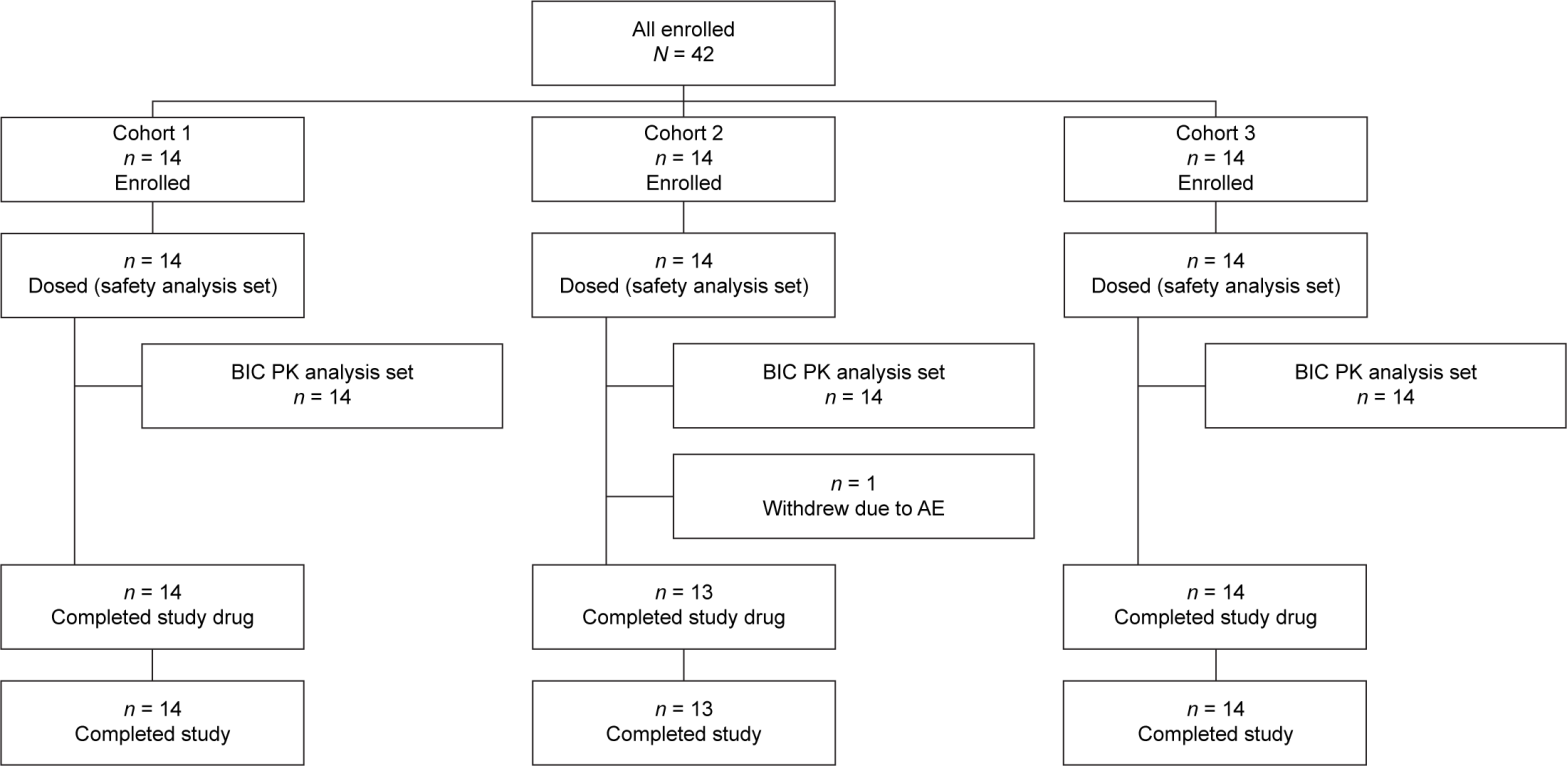
Disposition of study participants. AE, adverse event; BIC, bictegravir; PK, pharmacokinetic.

The study took place between April 4 and May 20, 2016, with all participants enrolled between April 13 and 16, 2016. A total of 42 individuals participated in the study (14 participants in each of Cohorts 1–3), received at least one dose of study drug, and were included in the safety and BIC PK analysis sets. Overall, 41 participants (98%) completed the study. One participant in Cohort 2 discontinued study drug because of an AE of Grade 2 urticaria (Fig. 1).

Baseline demographics and characteristics are shown in Table S1. Most participants were male (69%), White (71%), and of Hispanic/Latino ethnicity (69%); median age (quartile [Q]1, Q3) was 34 (29, 40) years. Participants had a median body mass index (Q1, Q3) of 26.6 (24.7, 28.5) kg/m^2^ and a median (Q1, Q3) estimated glomerular filtration rate of 118.7 (111.2, 134.7) mL/min.

### BIC PK outcomes

#### Cohort 1: Simultaneous co-administration (fasted)

**Fig 2:**
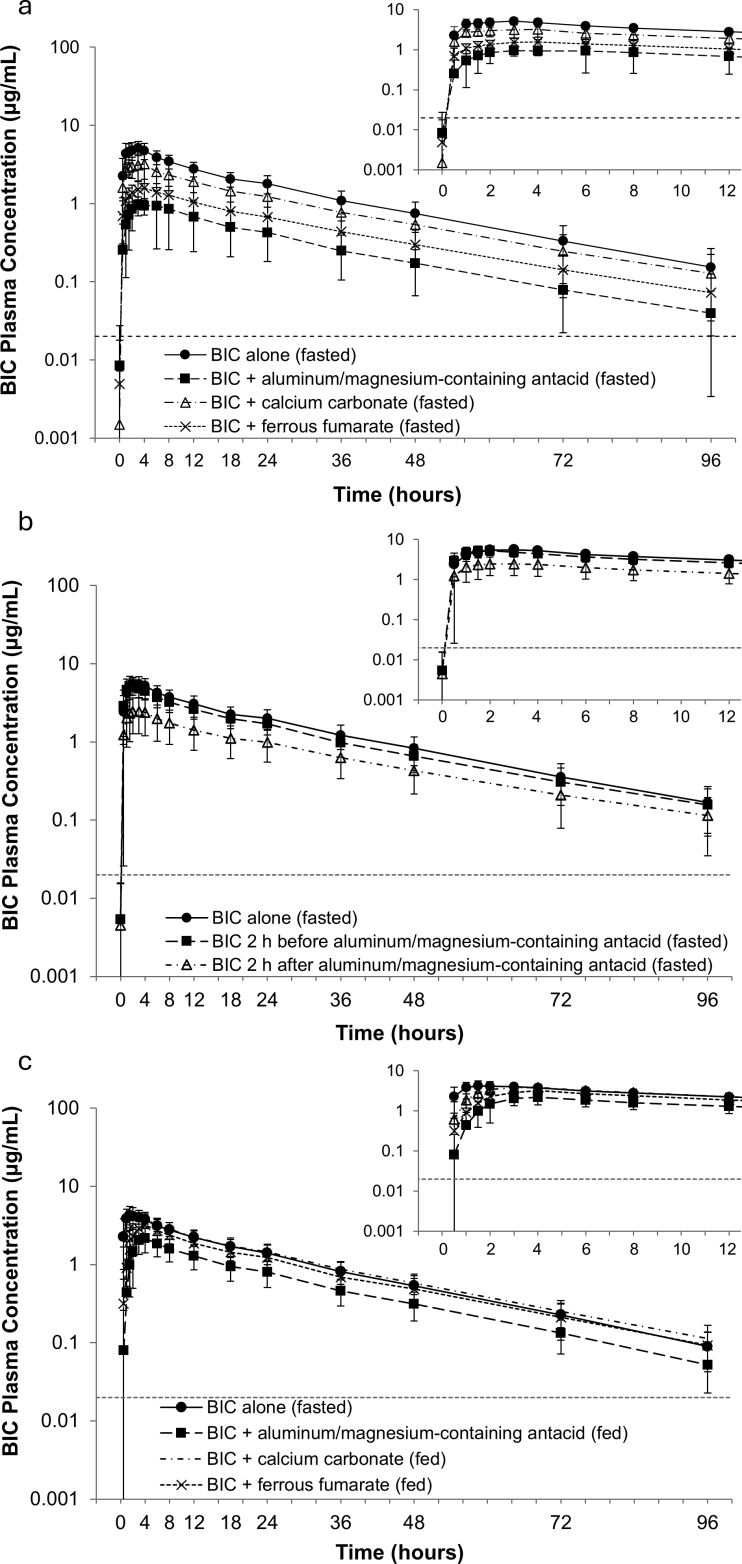
Mean (SD) BIC plasma concentration–time profiles (semi-logarithmic scale) by treatment (BIC PK analysis set). (**a**) Cohort 1, (**b**) Cohort 2, and (**c**) Cohort 3. Inset graphs show magnified views of the first 12 hours post-dose. Dashed lines indicate the lower level of quantification for BIC (0.020 µg/mL). B/F/TAF, bictegravir/emtricitabine/tenofovir alafenamide; BIC, bictegravir; PK, pharmacokinetic; SD, standard deviation.

Simultaneous co-administration of B/F/TAF with aluminum/magnesium-containing antacid, calcium carbonate, or ferrous fumarate resulted in reduced BIC exposure compared with B/F/TAF alone under fasted conditions (Tables 2 and 3; Fig. 2a). BIC AUC_inf_, C_24_, and C_max_ were reduced by 79%, 78%, and 80%, respectively, with aluminum/magnesium-containing antacid; by 33%, 35%, and 42%, respectively, with calcium carbonate; and by 63%, 63%, and 71%, respectively, with ferrous fumarate, compared with B/F/TAF alone. The 90% CIs of the GLSM ratios were outside the prespecified lack of DDI boundary of 70%–143%, indicating significant DDIs (Table 3).

**TABLE 2:** BIC plasma PK parameters by cohort (BIC PK analysis set)^*a*^

Parameter		Mean (%CV)		
Cohort 1—fasted, simultaneous co-administration	B/F/TAF + aluminum/magnesium-containing antacid (*N* = 14)	B/F/TAF + calcium carbonate (*N* = 14)	B/F/TAF + ferrous fumarate (*N* = 14)	B/F/TAF alone (*N* = 14)
AUC_inf_ (µg*h/mL)	28.0 (52.5)	85.0 (43.1)	46.1 (32.9)	121.9 (24.4)
C_max_ (µg/mL)	1.20 (52.0)	3.44 (36.9)	1.67 (27.1)	5.64 (18.8)
T_max_ (h)^*b*^	3.0 (2.0, 4.0)	1.8 (1.5, 4.0)	3.5 (3.0, 4.0)	2.0 (1.5, 3.0)
C_24_ (µg/mL)	0.43 (57.4)	1.22 (43.9)	0.67 (32.8)	1.80 (26.3)
AUC_last_ (µg*h/mL)	26.2 (52.8)	80.4 (42.5)	43.4 (31.4)	116.8 (22.8)
%AUC_exp_	5.9 (62.6)	5.3 (83.9)	5.5 (70.4)	3.8 (90.2)
T_1/2_ (h)^*b*^	20.2 (16.7, 25.2)	21.0 (17.6, 25.0)	21.9 (17.5, 25.2)	18.9 (14.6, 22.6)
CL/F (L/h)	2.18 (45.7)	0.68 (37.9)	1.19 (30.2)	0.43 (24.1)
V_Z_/F (L)	61.4 (27.9)	21.7 (48.8)	37.3 (27.6)	11.8 (18.3)

Cohort 2—fasted, staggered administration	B/F/TAF 2 hours before aluminum/magnesium-containing antacid (*N* = 13)	B/F/TAF 2 hours after aluminum/magnesium-containing antacid (*N* = 13)	B/F/TAF alone (*N* = 14)	
AUC_inf_ (µg*h/mL)	115.9 (30.3)	67.7 (47.0)	132.8 (27.0)	
C_max_ (µg/mL)	5.62 (22.7)	2.74 (48.3)	5.92 (16.5)	
T_max_ (h)^*b*^	1.5 (1.5, 2.0)	2.0 (1.5, 3.0)	3.0 (2.0, 3.0)	
C_24_ (µg/mL)	1.70 (27.9)	0.99 (44.0)	2.01 (28.3)	
AUC_last_ (µg*h/mL)	110.6 (28.8)	63.4 (45.8)	124.7 (28.0)	
%AUC_exp_	4.2 (49.4)	6.0 (48.7)	5.8 (148.5)	
T_1/2_ (h)^*b*^	21.8 (18.6, 24.2)	24.2 (20.1, 26.5)	20.8 (16.5, 22.8)	
CL/F (L/h)	0.47 (30.8)	0.91 (47.7)	0.41 (29.9)	
V_Z_/F (L)	14.4 (29.3)	31.2 (55.3)	11.2 (30.1)	

Cohort 3—fed, simultaneous co-administration	B/F/TAF + aluminum/magnesium-containing antacid (*N* = 14)	B/F/TAF + calcium carbonate (*N* = 14)	B/F/TAF + ferrous fumarate (*N* = 14)	B/F/TAF alone (fasted) (*N* = 14)
AUC_inf_ (µg*h/mL)	50.8 (34.8)	94.8 (21.2)	77.3 (24.8)	93.7 (27.2)
C_max_ (µg/mL)	2.45 (31.4)	4.11 (13.7)	3.49 (23.2)	4.70 (23.6)
T_max_ (h)^*b*^	3.5 (3.0, 4.0)	3.5 (2.0, 4.0)	4.0 (3.0, 4.0)	1.5 (1.5, 2.0)
C_24_ (µg/mL)	0.80 (36.4)	1.46 (22.1)	1.23 (25.0)	1.41 (29.7)
AUC_last_ (µg*h/mL)	49.2 (34.8)	91.5 (20.1)	74.5 (24.0)	91.2 (26.5)
%AUC_exp_	3.2 (31.8)	3.4 (42.5)	3.5 (39.6)	2.5 (38.9)
T_1/2_ (h)^*b*^	17.9 (16.9, 20.2)	18.9 (18.2, 21.6)	19.3 (18.2, 22.4)	18.0 (17.5, 19.9)
CL/F (L/h)	1.11 (37.8)	0.55 (21.7)	0.68 (20.5)	0.59 (41.1)
V_Z_/F (L)	29.1 (33.8)	15.5 (21.3)	19.3 (19.3)	15.4 (47.3)

^*a*^ %AUC_exp_, percentage of AUC extrapolated between AUC_last_ and AUC_inf_; AUC, area under the plasma concentration–time curve; AUC_inf_, AUC extrapolated to infinity; AUC_last_, AUC from time zero to the last quantifiable concentration; B/F/TAF, bictegravir/emtricitabine/tenofovir alafenamide; BIC, bictegravir; C_24_, plasma concentration at 24 hours; CL/F, apparent oral clearance; C_max_, maximum observed plasma concentration; %CV, percentage coefficient of variation; PK, pharmacokinetics; Q, quartile; T_max_, time of C_max_; T_1/2_, terminal elimination half-life; V_z_/F, apparent volume of distribution.

^*b*^ Median (Q1, Q3).

**TABLE 3:** Statistical comparison of BIC plasma PK parameters between treatments (BIC PK analysis set)^*a*^

Parameter	Test GLSM	Reference GLSM	%GLSM ratio (90% CI) (test/reference)
Cohort 1—fasted, simultaneous co-administration			
B/F/TAF with aluminum/magnesium-containing antacid	BIC + antacid (*N* = 14)	BIC alone (*N* = 14)	
AUC_inf_ (µg*h/mL)	25.2	118.6	21.2 (17.6, 25.7)
C_24_ (µg/mL)	0.38	1.74	21.9 (17.8, 27.0)
C_max_ (µg/mL)	1.10	5.54	19.9 (16.5, 24.0)

B/F/TAF with calcium carbonate	BIC + calcium carbonate (*N* = 14)	BIC alone (*N* = 14)	
AUC_inf_ (µg*h/mL)	79.1	118.6	66.7 (56.7, 78.4)
C_24_ (µg/mL)	1.13	1.74	64.9 (54.5, 77.3)
C_max_ (µg/mL)	3.23	5.54	58.3 (50.7, 67.0)

B/F/TAF with ferrous fumarate	BIC + ferrous fumarate (*N* = 14)	BIC alone (*N* = 14)	
AUC_inf_ (µg*h/mL)	44.0	118.6	37.1 (33.0, 41.8)
C_24_ (µg/mL)	0.64	1.74	36.9 (32.6, 41.8)
C_max_ (µg/mL)	1.61	5.54	29.1 (25.9, 32.7)

Cohort 2—fasted, staggered administration			
B/F/TAF 2 h before aluminum/magnesium-containing antacid	BIC + antacid (*N* = 14)	BIC alone (*N* = 14)	
AUC_inf_ (µg*h/mL)	111.1	128.1	86.7 (81.0, 92.8)
C_24_ (µg/mL)	1.65	1.93	85.5 (79.9, 91.4)
C_max_ (µg/mL)	5.46	5.84	93.4 (87.5, 99.7)

B/F/TAF 2 h after aluminum/magnesium-containing antacid	BIC + antacid (*N* = 14)	BIC alone (*N* = 14)	
AUC_inf_ (µg*h/mL)	61.1	128.1	47.7 (38.3, 59.4)
C_24_ (µg/mL)	0.90	1.93	46.6 (37.8, 57.6)
C_max_ (µg/mL)	2.43	5.84	41.5 (33.3, 51.8)

Cohort 3—fed, simultaneous co-administration			
B/F/TAF with aluminum/magnesium-containing antacid	BIC + antacid (*N* = 14)	BIC alone (*N* = 14)	
AUC_inf_ (µg*h/mL)	47.9	89.9	53.3 (44.2, 64.1)
C_24_ (µg/mL)	0.75	1.35	56.0 (46.2, 67.9)
C_max_ (µg/mL)	2.34	4.54	51.5 (42.7, 62.0)

B/F/TAF with calcium carbonate	BIC + calcium carbonate (*N* = 14)	BIC alone (*N* = 14)	
AUC_inf_ (µg*h/mL)	92.9	89.9	103.3 (89.0, 120.0)
C_24_ (µg/mL)	1.43	1.35	105.9 (89.8, 124.8)
C_max_ (µg/mL)	4.07	4.54	89.6 (77.8, 103.1)

B/F/TAF with ferrous fumarate	BIC + ferrous fumarate (*N* = 14)	BIC alone (*N* = 14)	
AUC_inf_ (µg*h/mL)	75.4	89.9	83.8 (74.1, 94.9)
C_24_ (µg/mL)	1.20	1.35	88.9 (78.1, 101.2)
C_max_ (µg/mL)	3.41	4.54	75.1 (64.8, 87.1)

^*a*^ AUC_inf_, area under the plasma concentration–time curve extrapolated to infinity; B/F/TAF, bictegravir/emtricitabine/tenofovir alafenamide; BIC, bictegravir; C_24_, plasma concentration at 24 hours; CI, confidence interval; C_max_, maximum observed plasma concentration; GLSM, geometric least-squares mean; h, hours; PK, pharmacokinetics.

The median time of C_max_ (T_max_) was delayed by 1 hour and 1.5 hours with aluminum/magnesium-containing antacid and ferrous fumarate, respectively, compared with B/F/TAF alone, but the median terminal elimination half-life (T_1/2_) was similar between treatments. The mean BIC apparent oral clearance (CL/F) and apparent volume of distribution (V_z_/F) for each treatment increased following simultaneous co-administration of aluminum/magnesium-containing antacid (404% and 421%, respectively), calcium carbonate (56% and 84%, respectively), and ferrous fumarate (174% and 216%, respectively) with B/F/TAF compared with B/F/TAF alone.

#### Cohort 2: Staggered administration of aluminum/magnesium-containing antacid (fasted)

Administration of B/F/TAF under fasted conditions 2 hours before aluminum/magnesium-containing antacid counteracted the interaction and resulted in BIC exposure similar to that seen with B/F/TAF alone (Tables 2 and 3; Fig. 2b). The 90% CIs of the GLSM ratios for BIC AUC_inf_, C_24_, and C_max_ were within the prespecified lack of DDI boundary, indicating a lack of significant impact on BIC exposure.

Administration of B/F/TAF 2 hours after aluminum/magnesium-containing antacid resulted in a less marked reduction in BIC exposure than when administered simultaneously under fasted conditions (52%, 53%, and 59% decreases in AUC_inf_, C_24_, and C_max_, respectively, compared with B/F/TAF alone; Tables 2 and 3; Fig. 2b). However, this was not sufficient to completely counteract the interaction, and the 90% CIs of the GLSM ratios for BIC AUC_inf_, AUC from time zero to the last quantifiable concentration (AUC_last_), and C_max_ still spanned the prespecified lack of DDI boundary.

The median T_max_ was delayed by 1.5 hours and 1 hour when B/F/TAF was administered 2 hours before and 2 hours after aluminum/magnesium-containing antacid, respectively, compared with B/F/TAF alone, but the median BIC T_1/2_ was similar between treatments. The mean BIC CL/F and V_Z_/F were generally similar following B/F/TAF administration 2 hours before aluminum/magnesium-containing antacid but were increased following administration 2 hours after aluminum/magnesium-containing antacid (124% and 180%, respectively) compared with B/F/TAF alone.

Since administration of B/F/TAF 2 hours before aluminum/magnesium-containing antacid administration was deemed sufficient to counteract the DDI effect, the fourth, adaptive cohort, assessing the effect of 4-hour staggered administration, was not initiated.

#### Cohort 3: Simultaneous co-administration (fed)

Simultaneous co-administration of B/F/TAF with calcium carbonate or ferrous fumarate under fed conditions generally resulted in BIC exposure similar to that seen with BIC alone under fasted conditions (Tables 2 and 3; Fig. 2c). The 90% CIs of the GLSM ratios for AUC_inf_, C_24_, and C_max_ were within the prespecified lack of DDI boundary, with the exception of the lower bound of the 90% CI for C_max_ with ferrous fumarate, which was slightly outside of this boundary.

Co-administration of B/F/TAF with aluminum/magnesium-containing antacid under fed conditions resulted in a less marked reduction in BIC exposure than when administered under fasted conditions (47%, 44%, and 49% decreases in AUC_inf_, C_24_, and C_max_, respectively, compared with B/F/TAF alone under fasted conditions; Tables 2 and 3; Fig. 2c). However, this was not sufficient to completely counteract the interaction, and the 90% CIs of the GLSM ratios for BIC AUC_inf_, C_24_, and C_max_ were still outside of the prespecified lack of DDI boundary.

The median T_max_ was delayed by 2 hours with simultaneous co-administration of B/F/TAF with aluminum/magnesium-containing antacid or calcium carbonate and by 2.5 hours with ferrous fumarate under fed conditions, compared with B/F/TAF alone under fasted conditions, but the median T_1/2_ was similar between treatments. The mean BIC CL/F and V_z_/F increased following co-administration of aluminum/magnesium-containing antacid with B/F/TAF under fed conditions (89% in both cases) but were similar for calcium carbonate and ferrous fumarate under fed conditions, compared with B/F/TAF alone under fasted conditions.

### Safety

AEs and laboratory abnormalities are shown in Table 4. Across all cohorts and treatments, there were no Grade 3 or 4 AEs, serious AEs, or deaths. The only AE considered related to B/F/TAF by the investigator was a single event of Grade 2 urticaria experienced by one participant in Cohort 2, which began 1 day after receiving B/F/TAF alone and resulted in study drug discontinuation. The participant was treated with diphenhydramine, and the event resolved on Day 7.

**TABLE 4:** AEs by cohort (safety analysis set)^*a*^

	Cohort 1—fasted, simultaneous co-administration				Cohort 2—fasted, staggered administration			Cohort 3—fed, simultaneous co-administration			
	B/F/TAF (*N* = 14)	Antacid^*b*^ (*N* = 14)	Calcium (*N* = 14)	Iron (*N* = 14)	B/F/TAF (*N* = 14)	Before antacid^*b*^ (*N* = 13)	After antacid^*b*^ (*N* = 13)	B/F/TAF^*c*^ (*N* = 14)	Antacid^*b*^ (*N* = 14)	Calcium (*N* = 14)	Iron (*N* = 14)
AEs											
Any AE	0	0	0	0	2 (14)	0	1 (8)	2 (14)	2 (14)	4 (29)	0
Constipation	0	0	0	0	0	0	0	1 (7)	1 (7)	4 (29)	0
Headache	0	0	0	0	1 (7)	0	1 (8)	0	0	0	0
Presyncope	0	0	0	0	0	0	0	1 (7)	0	0	0
Acne	0	0	0	0	0	0	0	0	1 (7)	0	0
Urticaria	0	0	0	0	1 (7)	0	0	0	0	0	0
Grade 3 or 4 AEs	0	0	0	0	0	0	0	0	0	0	0
Treatment-related AEs	0	0	0	0	1 (7)^*d*^	0	0	0	0	0	0
SAEs	0	0	0	0	0	0	0	0	0	0	0
AEs leading to permanent study drug discontinuation	0	0	0	0	1 (7)^*d*^	0	0	0	0	0	0
Death	0	0	0	0	0	0	0	0	0	0	0
Laboratory abnormalities											
Any grade	2 (14)	1 (7)	1 (7)	2 (14)	0	1 (8)	0	3 (21)	0	1 (7)	1 (7)
Grade 3 or 4	0	1 (7)^*e*^	0	0	0	0	0	0	0	0	0

^*a*^ Data represent the number (%) of participants. AE, adverse event; B/F/TAF, bictegravir/emtricitabine/tenofovir alafenamide; SAE, serious adverse event.

^*b*^ Aluminum/magnesium-containing antacid.

^*c*^ Administered under fasted conditions.

^*d*^ Grade 2 urticaria; the participant was treated with diphenhydramine, and the event resolved on Day 7. No post-treatment laboratory abnormalities were noted, including no change in eosinophil count.

^*e*^ Grade 3 hematuria in a female participant; no red blood cells on microscopic evaluation.

## DISCUSSION

PWH often take antacids to treat heartburn and gastroesophageal reflux disease (18) and calcium/iron supplements to improve bone health and treat anemia associated with HIV infection and ART (19, 20); therefore, it is important to understand the effects of these treatments on ART regimens to provide dosing recommendations and DDI mitigation strategies to minimize the risk of virologic failure and the development of INSTI resistance.

In this study, BIC exposure was reduced following simultaneous co-administration of B/F/TAF with aluminum/magnesium-containing antacid, calcium carbonate, or ferrous fumarate under fasted conditions. These effects were mitigated by staggering administration and/or administration under fed conditions (32).

The largest reduction in BIC exposure was observed upon simultaneous co-administration of BIC (as B/F/TAF) with aluminum/magnesium-containing antacid, which had the highest metal cation concentration tested in this study; an intermediate decrease was seen with ferrous fumarate, and the smallest decrease was seen with calcium carbonate. The 90% CIs of the GLSM ratios for all of these medications were outside of the prespecified lack of DDI boundary; therefore, all could be deemed to have significant DDIs. However, all available cumulative data for BIC efficacy, including the overall risk-benefit balance, were taken into consideration to inform clinical acceptability and contextualize the observed data. As BIC has been shown to have a long dissociation half-life (~163 hours) and a flat exposure–response curve for efficacy over a wide dose/exposure range (27–31), the scenarios for staggered administration and/or co-administration with a meal, as conveyed in the dosing recommendations, were deemed to have no clinically meaningful effect based on the magnitude of changes observed in the study. The observed reductions in BIC exposure are likely to be due to chelation of BIC by the metal cations, limiting BIC absorption (33). In support of this, both the mean BIC CL/F and V_z_/F were increased by a similar extent following co-administration of B/F/TAF with the aluminum/magnesium-containing antacid, or calcium or iron supplements, indicating that these changes are a result of reductions in bioavailability (F).

The effect of metal cations on BIC exposure was reduced when administration was staggered or under fed conditions. Administration of B/F/TAF 2 hours before aluminum/magnesium-containing antacid under fasted conditions mitigated the chelation effects seen with co-administration under fasted conditions. As the aluminum/magnesium-containing antacid had the highest metal cation content tested in this study, resulting in the greatest reduction in BIC exposure, it is likely that staggered administration would also mitigate the chelation effects of the calcium/iron-containing supplements.

In addition, co-administration of B/F/TAF with calcium carbonate and ferrous fumarate after a moderate-fat meal counteracted the chelation effects seen with co-administration under fasted conditions. The decrease in BIC exposure was less marked when B/F/TAF was simultaneously co-administered with the aluminum/magnesium-containing antacid after a moderate-fat meal compared with co-administration under fasted conditions*.* However, the 90% CIs of the GLSM ratios were still outside of the prespecified lack of DDI boundary, indicating that there was a significant effect on BIC exposure. As BIC has been shown to have a long dissociation half-life (~163 hours) and a flat exposure–response curve for efficacy over a wide dose/exposure range (27–31), this effect was not considered clinically meaningful based on the magnitude of the observed changes.

As seen in previous studies, B/F/TAF was well tolerated when administered alone (23, 34) and when given in combination with aluminum/magnesium-containing antacid, calcium carbonate, or ferrous fumarate.

Our findings suggest that staggering the administration of B/F/TAF and metal cation–containing antacids or calcium/iron-containing supplements and co-administration of B/F/TAF and calcium/iron-containing supplements with a meal results in BIC plasma exposure similar to when B/F/TAF is administered alone and could be a strategy to mitigate the effects of antacids and mineral supplements on BIC exposure.

Reductions in BIC exposure may be of particular relevance in pregnant women with HIV who, in addition to reduced BIC exposure associated with pregnancy (31), may be more likely to take antacids and metal cation–containing supplements than non-pregnant PWH. We have previously reported lower BIC exposure in pregnant women with HIV-1 receiving B/F/TAF than in non-pregnant adults with HIV-1 (3, 31). Therefore, as gastroesophageal reflux disease affects approximately two-thirds of pregnant women and calcium-containing antacids are the preferred medication (35), it is important to consider mitigation strategies for the concomitant use of B/F/TAF with antacids and mineral supplements in this population.

Like BIC, the mechanism of action of other INSTIs, such as raltegravir, dolutegravir, and elvitegravir, involves the binding of magnesium at the integrase active site (11), and thus, they are also susceptible to the chelating effects of metal cation–containing therapeutic products. Indeed, similar to our findings, previous studies have reported reductions in the exposure of all three of these INSTIs when administered with metal cation–containing therapeutic products (13–17). These studies have also shown that the DDI effects of aluminum/magnesium-containing antacids were counteracted when dolutegravir (15) and elvitegravir (16) were administered 2 hours after antacid treatment. In addition, the DDI effects of ferrous fumarate and calcium carbonate on dolutegravir were prevented when dolutegravir was administered 2 hours before ferrous fumarate and calcium carbonate or simultaneously under fed conditions (17). For this reason, staggered administration of INSTIs and metal cation–containing therapeutic products and/or administration with food is recommended (36–40). Effects of antacids on raltegravir PK may be compounded by the fact that, unlike other INSTIs, raltegravir absorption is pH dependent (14), and co-administration of raltegravir with aluminum-, magnesium-, and/or calcium carbonate–containing antacids is not recommended (40).

In light of this study’s findings and in conjunction with the established exposure–response relationship for BIC over a wide exposure range, as well as cumulative data on BIC efficacy, including its long dissociation half-life (27–31), current recommendations are that B/F/TAF is to be administered at least 2 hours before or 2 hours after taking aluminum/magnesium-containing antacids or calcium- or iron-containing supplements, or that they are to be co-administered with a meal. Following individual regulatory agency reviews and labeling discussions, these dosing recommendations, and regional variations thereof, have been incorporated into the region-specific labels for B/F/TAF (3, 41). In addition, findings from this study have been contextualized to inform pregnancy-related considerations, with the recommendations for pregnant individuals, where BIC exposures from B/F/TAF are lowered compared with non-pregnant individuals (31), that B/F/TAF is to be administered at least 2 hours before or 6 hours after taking aluminum/magnesium-containing antacids, or calcium- or iron-containing supplements, or that calcium- or iron-containing supplements are to be co-administered with a meal (3, 41).

The recommendation to separate administration of B/F/TAF and aluminum/magnesium-containing antacids/supplements or calcium- or iron-based medications/supplements by at least 6 hours was to mitigate further lowering of BIC exposure and was based on previous experience with other drugs, including dolutegravir (36) and cabotegravir (39) (other INSTIs) as well as ciprofloxacin (42) and delafloxacin (43). These drugs are known to bind to polyvalent cation–containing medications via similar mechanisms to those of BIC.

Limitations of this study include the potential risks of bias associated with the open-label study design. In addition, findings in participants without HIV may not be directly applicable to the target population; however, since disease status is not expected to significantly impact BIC PK, these data are likely to translate to PWH. Furthermore, this study looked at single doses of B/F/TAF and metal cation–containing therapeutic products but did not assess the effects of metal cations on BIC exposure when these products are taken more than once daily or over a prolonged period of time. However, as single doses are generally more sensitive in assessing the absorption phase, which is expected to be most impacted by chelation-type DDIs, therapeutic products taken more than once daily are unlikely to have any further impact on the exposure of once-daily BIC. In addition, given the median T_1/2_ of approximately 17 hours for BIC (3), the data are expected to be extrapolatable to steady-state dosing. Finally, the small participant number and short study timeframe may not be adequate to identify rare AEs.

### Conclusions

In conclusion, single doses of B/F/TAF, alone or combined with aluminum/magnesium-containing antacid, calcium carbonate, or ferrous fumarate, were well tolerated in this study. The effect of aluminum/magnesium-containing antacid on BIC exposure was prevented by administering 2 hours after B/F/TAF. Co-administration of B/F/TAF with a meal counteracted the chelating effects of calcium carbonate and ferrous fumarate; in addition, the chelating effect of aluminum/magnesium-containing antacid was also reduced under fed conditions. These results support the recommendations that B/F/TAF is to be administered at least 2 hours before or 2 hours after taking aluminum/magnesium-containing antacids or calcium- or iron-containing supplements, or that they are to be co-administered with a meal. Furthermore, findings from this study were contextualized to inform pregnancy-related considerations in labeling, where additional staggering of B/F/TAF at least 6 hours after taking metal cation–containing medications or supplements is recommended to minimize reductions in BIC exposure.

## Data Availability

Gilead Sciences shares anonymized individual patient data upon request or as required by law or regulation with qualified external researchers on the basis of submitted curriculum vitae and reflecting non-conflict of interest. The request proposal must also include a statistician. Approval of such requests is at the discretion of Gilead Sciences and is dependent on the nature of the request, the merit of the research proposed, the availability of the data, and the intended use of the data. Data requests should be sent to datarequest@gilead.com.

## SUPPLEMENTAL MATERIAL

Supplemental material aac.00781-25-s0001.pdf Table S1.

ASM does not own the copyrights to Supplemental Material that may be linked to, or accessed through, an article. The authors have granted ASM a non-exclusive, world-wide license to publish the Supplemental Material files. Please contact the corresponding author directly for reuse.

## References

[1] Department of Health and Human Services (DHHS). 2024. Guidelines for the use of antiretroviral agents in adults and adolescents with HIV. Available from: https://clinicalinfo.hiv.gov/sites/default/files/guidelines/documents/adult-adolescent-arv/guidelines-adult-adolescent-arv.pdf. Retrieved 19 Sep 2025.

[2] European AIDS Clinical Society. 2024. Guidelines version 12.1. Available from: https://eacs.sanfordguide.com. Retrieved 19 Sep 2025.

[3] Gilead Sciences Ltd. 2025. Biktarvy. Available from: https://www.medicines.org.uk/emc/product/9313. Retrieved 11 Aug 2025.

[4] Molina JM, Ward D, Brar I, Mills A, Stellbrink HJ, López-Cortés L, Ruane P, Podzamczer D, Brinson C, Custodio J, Liu H, Andreatta K, Martin H, Cheng A, Quirk E. 2018. Switching to fixed-dose bictegravir, emtricitabine, and tenofovir alafenamide from dolutegravir plus abacavir and lamivudine in virologically suppressed adults with HIV-1: 48 week results of a randomised, double-blind, multicentre, active-controlled, phase 3, non-inferiority trial. Lancet HIV 5:e357–e365. doi:10.1016/S2352-3018(18)30092-4 29925489

[5] Orkin C, DeJesus E, Sax PE, Arribas JR, Gupta SK, Martorell C, Stephens JL, Stellbrink H-J, Wohl D, Maggiolo F, Thompson MA, Podzamczer D, Hagins D, Flamm JA, Brinson C, Clarke A, Huang H, Acosta R, Brainard DM, Collins SE, Martin H, GS-US-380-1489, GS-US-380-1490 study investigators. 2020. Fixed-dose combination bictegravir, emtricitabine, and tenofovir alafenamide versus dolutegravir-containing regimens for initial treatment of HIV-1 infection: week 144 results from two randomised, double-blind, multicentre, phase 3, non-inferiority trials. Lancet HIV 7:e389–e400. doi:10.1016/S2352-3018(20)30099-0 32504574

[6] Sax PE, Rockstroh JK, Luetkemeyer AF, Yazdanpanah Y, Ward D, Trottier B, Rieger A, Liu H, Acosta R, Collins SE, Brainard DM, Martin H, GS-US-380–4030 Investigators. 2021. Switching to bictegravir, emtricitabine, and tenofovir alafenamide in virologically suppressed adults with human immunodeficiency virus. Clin Infect Dis 73:e485–e493. doi:10.1093/cid/ciaa988 32668455 PMC8282313

[7] Stellbrink H-J, Arribas JR, Stephens JL, Albrecht H, Sax PE, Maggiolo F, Creticos C, Martorell CT, Wei X, Acosta R, Collins SE, Brainard D, Martin H. 2019. Co-formulated bictegravir, emtricitabine, and tenofovir alafenamide versus dolutegravir with emtricitabine and tenofovir alafenamide for initial treatment of HIV-1 infection: week 96 results from a randomised, double-blind, multicentre, phase 3, non-inferiority trial. Lancet HIV 6:e364–e372. doi:10.1016/S2352-3018(19)30080-3 31068272

[8] Ambrosioni J, Rojas Liévano J, Berrocal L, Inciarte A, de la Mora L, González-Cordón A, Martínez-Rebollar M, Laguno M, Torres B, Ugarte A, Chivite I, Leal L, de Lazzari E, Miró JM, Blanco JL, Martinez E, Mallolas J. 2022. Real-life experience with bictegravir/emtricitabine/tenofovir alafenamide in a large reference clinical centre. J Antimicrob Chemother 77:1133–1139. doi:10.1093/jac/dkab481 35040990

[9] Mounzer K, Brunet L, Fusco JS, Mcnicholl IR, Diaz Cuervo H, Sension M, Mccurdy L, Fusco GP. 2022. Advanced HIV infection in treatment-naive individuals: effectiveness and persistence of recommended 3-drug regimens. Open Forum Infect Dis 9:ofac018. doi:10.1093/ofid/ofac018 35169590 PMC8842315

[10] Mounzer K, Slim J, Ramgopal M, Hedgcock M, Bloch M, Santana J, Mendes I, Guo Y, Arora P, Montezuma-Rusca JM, Martin H, Sklar P, Baeten J, Segal-Maurer S. 2024. Phase II study of switch to daily BIC + LEN in individuals on a multitablet HIV treatment regimen (Poster 642). Presented at: Conference on Retroviruses and Opportunistic Infections (CROI). Denver, CO, USA

[11] Jóźwik IK, Passos DO, Lyumkis D. 2020. Structural biology of HIV integrase strand transfer inhibitors. Trends Pharmacol Sci 41:611–626. doi:10.1016/j.tips.2020.06.003 32624197 PMC7429322

[12] Scarsi KK, Havens JP, Podany AT, Avedissian SN, Fletcher CV. 2020. HIV-1 integrase inhibitors: a comparative review of efficacy and safety. Drugs (Abingdon Engl) 80:1649–1676. doi:10.1007/s40265-020-01379-9 PMC7572875 32860583

[13] Kiser JJ, Bumpass JB, Meditz AL, Anderson PL, Bushman L, Ray M, Predhomme JA, Rower J, Mawhinney S, Brundage R. 2010. Effect of antacids on the pharmacokinetics of raltegravir in human immunodeficiency virus-seronegative volunteers. Antimicrob Agents Chemother 54:4999–5003. doi:10.1128/AAC.00636-10 20921313 PMC2981249

[14] Krishna R, East L, Larson P, Valiathan C, Butterfield K, Teng Y, Hernandez-Illas M. 2016. Effect of metal-cation antacids on the pharmacokinetics of 1200 mg raltegravir. J Pharm Pharmacol 68:1359–1365. doi:10.1111/jphp.12632 27671833

[15] Patel P, Song I, Borland J, Patel A, Lou Y, Chen S, Wajima T, Peppercorn A, Min SS, Piscitelli SC. 2011. Pharmacokinetics of the HIV integrase inhibitor S/GSK1349572 co-administered with acid-reducing agents and multivitamins in healthy volunteers. J Antimicrob Chemother 66:1567–1572. doi:10.1093/jac/dkr139 21493648

[16] Ramanathan S, Mathias A, Wei X, Shen G, Koziara J, Cheng A, Kearney BP. 2013. Pharmacokinetics of once-daily boosted elvitegravir when administered in combination with acid-reducing agents. J Acquir Immune Defic Syndr 64:45–50. doi:10.1097/QAI.0b013e31829ecd3b 23774876

[17] Song I, Borland J, Arya N, Wynne B, Piscitelli S. 2015. Pharmacokinetics of dolutegravir when administered with mineral supplements in healthy adult subjects. J Clin Pharmacol 55:490–496. doi:10.1002/jcph.439 25449994 PMC4407950

[18] van Lunzen J, Liess H, Arastéh K, Walli R, Daut B, Schürmann D. 2007. Concomitant use of gastric acid-reducing agents is frequent among HIV-1-infected patients receiving protease inhibitor-based highly active antiretroviral therapy. HIV Med 8:220–225. doi:10.1111/j.1468-1293.2007.00456.x 17461849

[19] Lopes KG, Farinatti P, Lopes G de O, Paz GA, Bottino DA, Oliveira RB de, Bouskela E, Borges JP. 2021. Muscle mass, strength, bone mineral density and vascular function in middle-aged people living with HIV vs. age-matched and older controls. Braz J Infect Dis 25:101654. doi:10.1016/j.bjid.2021.101654 34826379 PMC9392186

[20] Abioye AI, Andersen CT, Sudfeld CR, Fawzi WW. 2020. Anemia, iron status, and HIV: a systematic review of the evidence. Adv Nutr 11:1334–1363. doi:10.1093/advances/nmaa037 32383731 PMC7490171

[21] Yetley EA. 2007. Multivitamin and multimineral dietary supplements: definitions, characterization, bioavailability, and drug interactions. Am J Clin Nutr 85:269S–276S. doi:10.1093/ajcn/85.1.269S 17209208

[22] Cockcroft DW, Gault MH. 1976. Prediction of creatinine clearance from serum creatinine. Nephron 16:31–41. doi:10.1159/000180580 1244564

[23] Gallant J, Lazzarin A, Mills A, Orkin C, Podzamczer D, Tebas P, Girard PM, Brar I, Daar ES, Wohl D, Rockstroh J, Wei X, Custodio J, White K, Martin H, Cheng A, Quirk E. 2017. Bictegravir, emtricitabine, and tenofovir alafenamide versus dolutegravir, abacavir, and lamivudine for initial treatment of HIV-1 infection (GS-US-380-1489): a double-blind, multicentre, phase 3, randomised controlled non-inferiority trial. Lancet 390:2063–2072. doi:10.1016/S0140-6736(17)32299-7 28867497

[24] Drugs.com. 2025. Aluminum hydroxide / magnesium hydroxide / simethicone dosage. Available from: https://www.drugs.com/dosage/aluminum-hydroxide-magnesium-hydroxide-simethicone.html. Retrieved 19 Sep 2025.

[25] Drugs.com. 2025. Calcium carbonate dosage. Available from: https://www.drugs.com/dosage/calcium-carbonate.html. Retrieved 19 Sep 2025.

[26] Drugs.com. 2025. Ferrous fumarate dosage. Available from: https://www.drugs.com/dosage/ferrous-fumarate.html. Retrieved 19 Sep 2025.

[27] Arora P, Zhang H, Hindman J, Liu H, Girish S, Davis C, Palaparthy R. 2024. Pharmacokinetics, safety, and efficacy study in pregnancy and existing cumulative data/evidence to support clinical use and labeling of B/F/TAF in pregnant women with HIV (Poster 26). Presented at: International Workshop on Clinical Pharmacology of HIV. Liverpool, UK

[28] Gallant JE, Thompson M, DeJesus E, Voskuhl GW, Wei X, Zhang H, White K, Cheng A, Quirk E, Martin H. 2017. Antiviral activity, safety, and pharmacokinetics of bictegravir as 10-day monotherapy in HIV-1-infected adults. J Acquir Immune Defic Syndr 75:61–66. doi:10.1097/QAI.0000000000001306 28196003 PMC5389589

[29] Center for Drug Evaluation and Research. 2018. BIKTARVY NDA 210251 uni‐review. Available from: https://www.accessdata.fda.gov/drugsatfda_docs/nda/2018/210251Orig1s000MultidisciplineR.pdf. Retrieved 17 Jan 2025.

[30] White KL, Osman N, Cuadra-Foy E, Brenner BG, Shivakumar D, Campigotto F, Tsiang M, Morganelli PA, Novikov N, Lazerwith SE, Jin H, Niedziela-Majka A. 2023. Long dissociation of bictegravir from HIV-1 integrase-DNA complexes. Antimicrob Agents Chemother 65:e02406-20. doi:10.1128/AAC.02406-20 33649107 PMC8092912

[31] Zhang H, Hindman JT, Lin L, Davis M, Shang J, Xiao D, Avihingsanon A, Arora P, Palaparthy R, Girish S, Marathe DD. 2024. A study of the pharmacokinetics, safety, and efficacy of bictegravir/emtricitabine/tenofovir alafenamide in virologically suppressed pregnant women with HIV. AIDS 38:F1–F9. doi:10.1097/QAD.0000000000003783 37939141 PMC10715703

[32] Mathias A, Lutz JD, West SK, Xiao D, Chuck SK, Martin H, Quirk E, Kearney BP. Pharmacokinetics of bictegravir in combination with polyvalent cation-containing antacids and supplements (Poster P260). Presented at: HIV Drug Therapy Glasgow. Glasgow, UK

[33] Lu CH, Bednarczyk EM, Catanzaro LM, Shon A, Xu JC, Ma Q. 2021. Pharmacokinetic drug interactions of integrase strand transfer inhibitors. Curr Res Pharmacol Drug Discov 2:100044. doi:10.1016/j.crphar.2021.100044 34909672 PMC8663927

[34] Sax PE, DeJesus E, Crofoot G, Ward D, Benson P, Dretler R, Mills A, Brinson C, Peloquin J, Wei X, White K, Cheng A, Martin H, Quirk E. 2017. Bictegravir versus dolutegravir, each with emtricitabine and tenofovir alafenamide, for initial treatment of HIV-1 infection: a randomised, double-blind, phase 2 trial. Lancet HIV 4:e154–e160. doi:10.1016/S2352-3018(17)30016-4 28219610

[35] Altuwaijri M. 2022. Evidence-based treatment recommendations for gastroesophageal reflux disease during pregnancy: a review. Medicine (Abingdon) 101:e30487. doi:10.1097/MD.0000000000030487 PMC9439837 36107559

[36] ViiV Healthcare. 2022. Tivicay. Available from: https://gskpro.com/content/dam/global/hcpportal/en_US/Prescribing_Information/Tivicay/pdf/TIVICAY-PI-PIL-IFU.PDF. Retrieved 16 May 2025.

[37] Gilead Sciences, Inc. 2021. Stribild. Available from: https://www.gilead.com/~/media/Files/pdfs/medicines/hiv/stribild/stribild_pi.pdf. Retrieved 19 Sep 2025.

[38] ViiV Healthcare. 2024. Dovato. Available from: https://gskpro.com/content/dam/global/hcpportal/en_US/Prescribing_Information/Dovato/pdf/DOVATO-PI-PIL.PDF. Retrieved 19 Sep 2025.

[39] ViiV Healthcare. 2025. Vocabria. Available from: https://gskpro.com/content/dam/global/hcpportal/en_US/Prescribing_Information/Vocabria/pdf/VOCABRIA-PI-PIL.PDF. Retrieved 28 Aug 2025.

[40] Merck Sharp & Dohme LLC. 2022. Isentress. Available from: https://www.merck.com/product/usa/pi_circulars/i/isentress/isentress_pi.pdf. Retrieved 5 Mar 2024.

[41] Gilead Sciences Ireland UC. 2025. Biktarvy. Available from: https://www.ema.europa.eu/en/documents/product-information/biktarvy-epar-product-information_en.pdf. Retrieved 8 Aug 2025.

[42] Bayer HealthCare Pharmaceuticals Inc. 2024. Cipro. Available from: https://www.accessdata.fda.gov/drugsatfda_docs/label/2024/019537s095,020780s050lbl.pdf. Retrieved 28 Aug 2025.

[43] Melinta Therapeutics, LLC. 2021. Baxdela. Available from: https://baxdela.com/docs/baxdela-prescribing-information.pdf. Retrieved 28 Aug 2025.

