# Supplementary material for: The effect of antacid and mineral supplements on bictegravir pharmacokinetics: results from a Phase 1, open-label, drug–drug interaction study
Source: Antimicrob Agents Chemother. 2025 Nov 28;70(1):e00781-25. doi: 10.1128/aac.00781-25 (PMC12777568; doi:10.1128/aac.00781-25)
Supplement: Supplemental material — Table S1. [file aac.00781-25-s0001.pdf]

Supplemental Material

Inclusion/exclusion criteria

- Participants who met all of the following criteria were eligible for participation in the study:
    - Participants had to have the ability to understand and sign a written informed consent form, which was obtained prior to initiation of study procedures
    - Participants had to be between 18 and 45 years of age, inclusive, at screening
    - Participants had to be nonsmokers. The use of nicotine or nicotine-containing products was discontinued 90 days prior to the first dose of study drug
    - The calculated body mass index had to be from 19 to 30 kg/m^2, inclusive, at screening
    - Estimated glomerular filtration rate using the Cockcroft-Gault method (24) had to be ≥ 90 mL/min, based on serum creatinine and actual body weight as measured at screening
    - Female participants of childbearing potential had to have had a negative serum pregnancy test at screening and clinic admission

- Female participants who utilized hormonal contraceptive as one of their birth control methods must have used the same method for at least 3 months prior to study dosing
- Male and female participants of childbearing potential who engaged in heterosexual intercourse agreed to use protocol-specified method(s) of contraception
- Male participants refrained from sperm donation from clinic admission, throughout the study period
- Participants did not donate blood within 56 days of study entry or plasma within 7 days of study entry and refrained from blood donation from clinic admission, throughout the study period and for at least 30 days following the last dose of study drug
- Participants must, in the opinion of the investigator, have been in good health based upon medical history and physical examination, including vital signs
- Screening laboratory evaluations (hematology, fasting lipids, chemistry, and urinalysis) had to be within the normal range of the local laboratory's reference ranges, unless the results were determined by the investigator to have no clinical significance
- Alanine aminotransferase, aspartate aminotransferase, alkaline phosphatase, and total bilirubin had to be normal or below the upper limit of normal at screening

- Electrocardiogram (ECG) had to be normal, or if the ECG had abnormalities, they had to be considered clinically insignificant by the investigator
- Participants had to be willing and able to comply with all study requirements

- Participants who met any of the following criteria were not eligible for participation in the study:
  - Pregnant or lactating participants
  - Participants with any serious or active medical or psychiatric illness (including depression) that, in the opinion of the investigator, could interfere with participant treatment, assessment, or compliance with the protocol. This included renal, cardiac, hematological, hepatic, pulmonary (including chronic asthma), endocrine (including diabetes), central nervous, gastrointestinal (including an ulcer), vascular, metabolic (thyroid disorders, adrenal disease), immunodeficiency disorders, active infection, or malignancy that were clinically significant or required treatment
  - Participants who had received any investigational compound within 30 days prior to study dosing
  - Participants with current alcohol or substance abuse judged by the investigator to potentially interfere with participant compliance or safety
  - Participants with a positive test for drugs of abuse, including alcohol at screening or on Day −1/check-in

- Participants with a positive test result for HIV-1 antibody, hepatitis B surface antigen, or hepatitis C antibody
- Participants with poor venous access that limited phlebotomy
- Participants who had taken any prescription medications or over-the-counter medications, including herbal products and antacids, within 28 days prior to start of study drug dosing, with the exception of vitamins and/or acetaminophen and/or ibuprofen and/or hormonal contraceptive medications
- Participants who had been treated with systemic steroids, immunosuppressant therapies, or chemotherapeutic agents within 3 months prior to screening or expected to receive these agents during the study (e.g. corticosteroids, immunoglobulins, and other immune- or cytokine-based therapies)
- Participants with significant drug sensitivity or drug allergy (such as anaphylaxis or hepatotoxicity)
- Participants with known hypersensitivity to the study drugs or to formulation excipients
- Participants with significant serious skin disease, such as but not limited to rash, food allergy, eczema, psoriasis, or urticaria
- Participants with liver disease, including Gilbert’s disease

- Participants with severe peptic ulcer disease, gastroesophageal reflux disease, or other gastric acid hypersecretory conditions that required prolonged (> 6 months) medical treatment
- Participants with medical or surgical treatment that permanently altered gastric absorption (e.g. gastric or intestinal surgery); a history of cholecystectomy was not exclusionary
- Participants with syncope, palpitations, or unexplained dizziness
- Participants with significant cardiac disease (including history of myocardial infarction based on ECG and/or clinical history, any history of ventricular tachycardia, congestive heart failure, or dilated cardiomyopathy with left ventricular ejection fraction < 40%), a family history of long QT syndrome, or unexplained death in an otherwise healthy individual between the ages of 1 and 30 years
- Participants with the presence or history of cardiovascular disease, cardiomyopathy, and/or cardiac conduction abnormalities
- Participants with an implanted defibrillator or pacemaker
- Participants who were unable to comply with study requirements or who were otherwise believed, by the investigator, to be inappropriate for study participation

Table S1. Baseline demographics and characteristics of participants (safety analysis set)

	Cohort 1 (N = 14)	Cohort 2 (N = 14)	Cohort 3 (N = 14)	Total (N = 42)
Age,[a] median (Q1, Q3), years	32 (30, 35)	29 (27, 35)	41 (33, 42)	34 (29, 40)
Males, n (%)	10 (71)	10 (71)	9 (64)	29 (69)
Females, n (%)	4 (29)	4 (29)	5 (36)	13 (31)
Race, n (%)				
White	8 (57)	12 (86)	10 (71)	30 (71)
Black/African American	6 (43)	2 (14)	4 (29)	12 (29)
Ethnicity, n (%)				
Hispanic or Latino	8 (57)	12 (86)	9 (64)	29 (69)
Not Hispanic or Latino	6 (43)	2 (14)	5 (36)	13 (31)
BMI, median (Q1, Q3), kg/m^2	26.1 (24.7, 27.6)	25.6 (22.8, 28.0)	28.1 (26.5, 28.8)	26.6 (24.7, 28.5)
eGFR[b] median (Q1, Q3), mL/min	118.6 (111.2, 134.7)	129.3 (112.8, 139.6)	117.5 (107.1, 124.0)	118.7 (111.2, 134.7)

[a]Age was calculated in years from the date of first dose of study drug. [b]Calculated by Cockcroft-Gault formula

BMI, body mass index; eGFR, estimated glomerular filtration rate; Q, quartile
